# Predictive value of pre-arrest albumin level with GO-FAR score in patients with in-hospital cardiac arrest

**DOI:** 10.1038/s41598-021-90203-9

**Published:** 2021-05-20

**Authors:** Seok-In Hong, Youn-Jung Kim, Yeon Joo Cho, Jin Won Huh, Sang-Bum Hong, Won Young Kim

**Affiliations:** 1 Department of Emergency Medicine, University of Ulsan College of Medicine, Asan Medical Center, Seoul, 05505 South Korea; 2 Department of Emergency Medicine, Kyungpook National University School of Medicine, Daegu, 41404 South Korea; 3 Department of Pulmonary and Critical Care Medicine, University of Ulsan College of Medicine, Asan Medical Center, Seoul, 05505 South Korea

**Keywords:** Prognostic markers, Cardiovascular diseases

## Abstract

We investigated whether combining the pre-arrest serum albumin level could improve the performance of the Good Outcome Following Attempted Resuscitation (GO-FAR) score for predicting neurologic outcomes in in-hospital cardiac arrest patients. Adult patients who were admitted to a tertiary care hospital between 2013 and 2017 were assessed. Their pre-arrest serum albumin levels were measured within 24 h before the cardiac arrest. According to albumin levels, the patients were divided into quartiles and were assigned 1, 0, 0, and, − 2 points. Patients were allocated to the derivation (n = 419) and validation (n = 444) cohorts. The proportion of favorable outcome increased in a stepwise manner across increasing quartiles (p for trend < 0.018). Area under receiver operating characteristic curve (AUROC) of the albumin-added model was significantly higher than that of the original GO-FAR model (0.848 vs. 0.839; *p* = 0.033). The results were consistent in the validation cohort (AUROC 0.799 vs. 0.791; *p* = 0.034). Net reclassification indices of the albumin-added model were 0.059 (95% confidence interval [CI] − 0.037 to 0.094) and 0.072 (95% CI 0.013–0.132) in the derivation and validation cohorts, respectively. An improvement in predictive performance was found by adding the ordinal scale of pre-arrest albumin levels to the original GO-FAR score.

## Introduction

The number of patients with in-hospital cardiac arrest (IHCA) has increased. Approximately 1 out of every 339 hospitalized adults^1^ has IHCA. Although the survival rate of IHCA patients has increased to 22.3% due to advances in cardiopulmonary resuscitation (CPR), the probability of a favorable outcome after CPR remains poor^2^. Many of these IHCA survivors require prolonged care, which is burdensome to the society and families. Therefore, accurate prediction of favorable neurological survival after IHCA could provide critical information for physicians and family members, and could help with appropriate treatment decisions^3–5^. Further, these medical decisions should be made before cardiac arrest to respect the patient’s wishes.

The Good Outcome Following Attempted Resuscitation (GO-FAR) score is a summed score comprising 13 pre-arrest variables with values ranging from − 15 to 11 points (Supplementary Table S1). It is used to predict the likelihood of neurologically intact survival. Although they are relatively an old data set now, the GO-FAR score was developed from a large database, which included 51,240 IHCA patients between 2007 and 2009^6^. The neurologically intact survival in the GO-FAR score was defined as Cerebral Performance Category (CPC) score 1; however, in many studies investigating outcomes of cardiac arrests, CPC scores 1 and 2 are considered favorable outcomes^7,8^.

Low serum albumin levels are associated with inflammation, malnutrition, and old age. Hypoalbuminemia, i.e. albumin level less than 3.5 g/dL is reported in up to 50% of critically ill patients^9^. Albumin plays an important role in many physiological mechanisms^10,11^ and has a strong prognostic value of mortality in various conditions such as sepsis, severe burns, and major surgeries^12–14^. Some studies showed that reduced albumin levels at achieved return of spontaneous circulation were independently associated with increased mortality^15^ among patients with out-of-hospital cardiac arrest (OHCA). A recent prospective observational study suggested that high serum albumin levels were independently associated with favorable neurologic outcomes in OHCA patients^16^. Despite the association between hypoalbuminemia and adverse outcomes of various critical illnesses, data on the prognostic value of albumin levels for outcomes among IHCA patients (particularly those measured before cardiac arrest) are limited.

Considering albumin levels in critically ill patients, we hypothesized that predictive power of the original GO-FAR score for neurologic outcomes in IHCA patients will improve by adding the values of pre-arrest albumin levels. Albumin levels of patients are assessed during routine laboratory tests. Thus, the values can be obtained and added to the GO-FAR model easily. This study aimed to evaluate the predictive value of pre-arrest albumin level and to compare the predictive performance for favorable neurologic outcomes between the original GO-FAR model and the albumin-added GO-FAR model.

## Methods

### Study design and population

This observational study was conducted at the Asan Medical Center, a 2700-bed tertiary care hospital in Seoul, Korea. We used a prospectively enrolled registry of IHCA patients to enroll consecutive adult patients (> 18 years) admitted to the hospital between 2013 and 2017. The IHCA patients were enrolled to the registry on the date of cardiac arrest and the study period was set based on enroll time. We identified the patients whose serum albumin levels were measured before cardiac arrests. Patients who had insufficient data for calculating the GO-FAR score or whose pre-arrest albumin levels were not measured were excluded. The ethics committee of Asan Medical Center approved the study protocols and waived the requirement of informed consent. Personal information of all patients was anonymized and removed before analysis. The study was conducted in accordance with the principles of the Declaration of Helsinki.

### IHCA registry

The medical emergency team (MET) system has been employed at Asan Medical Center since March 2008^17^. As members of the cardiac arrest team, the MET manages all IHCA patients even after CPR. After CPR, nurses fill the online CPR registry within 24 h. The data in the IHCA registry is reviewed and validated by the MET and the resuscitation committee; it includes demographic history (e.g. age, sex, medical history, and diagnosis at hospital visits), resuscitation profiles (e.g. location and initial cardiac rhythm when cardiac arrest was identified), critical interventions at the time of cardiac arrest, defibrillation during resuscitation, time required to defibrillate, and outcomes (e.g. survival and neurologic status).The CPC score was used to measure the neurologic status after cardiac arrest^7^. For this, patients were classified into five categories: 1 (good cerebral performance: conscious, able to work, might have mild neurologic or psychologic deficit); 2 (moderate cerebral disability: conscious, sufficient cerebral function for independent activities of daily life); 3 (severe cerebral disability: conscious, dependent on others for daily support because of impaired brain function); 4 (coma or vegetative state: any degree of coma without brain death criteria); and 5 (brain death).

### Data collection and statistical analyses

Neurologic outcomes after IHCA were retrieved from the registry. Albumin levels were measured within 24 h of IHCA, and the closest result to cardiac arrest was selected if albumin levels were measured more than once. Patients were considered to have active cancer if they presented tumor burden or they underwent chemo- or radiotherapy within 3 months of cardiac arrest.

The primary end point of this study was discharge with favorable neurologic outcome, defined as a CPC score of 1 or 2.

Baseline characteristics of included patients were presented as numbers and percentages for categorical variables and medians (interquartile ranges) for continuous variables. Differences between groups were analyzed using Fisher’s exact test (categorical variables) and Mann–Whitney U test (continuous variables).

Based on serum albumin levels, patients were divided into quartiles (Q1–Q4) as Q1 (< 2.1 g/dL), Q2 (2.1–2.5 g/dL), Q3 (2.6–3.0 g/dL), and Q4 (> 3.0 g/dL). We categorized the quartiles into three ordinal scales (1, 0, and − 2) according to the odds ratio (OR) in logistic analysis for favorable neurologic outcome: 1 was assigned to Q1, 0 was assigned to Q2 and Q3, and − 2 was assigned to Q4. The association between albumin quartiles and neurologic outcomes was examined using univariable and multivariable logistic regression analyses, and the results were presented as ORs and 95% confidence intervals (CIs). Potential confounders were also examined, and variables with *p* values less than 0.1 in univariable analyses were selected for multivariable analysis. Two-tailed *p* values less than 0.05 were considered statistically significant. Improvement of the model for prediction of neurologic outcomes were analyzed through calculating the areas under receiver operating characteristic curve (AUROCs), and statistical differences between curves were examined using Delong’s test. All statistical analyses were performed using IBM SPSS Statistics for Windows (version 21.0, IBM Corp., Armonk, NY, USA) or R (version 4.02, The R Foundation for Statistical Computing, Vienna, Austria).

## Results

A total of 1011 patients with IHCA was reviewed, and after excluding 148 patients without measured values of pre-arrest albumin levels, remaining 863 patients were included. They were divided into the derivation (from March 2013 to February 2015; n = 419) and validation cohorts (from March 2015 to March 2017; n = 444) (Supplementary Fig. S1). Overall, the median age was 64.0 years (54.0–74.0 years) and 127 patients (14.7%) were discharged with favorable neurologic outcomes.

### Derivation

Baseline clinical characteristics of the derivation cohort are presented in Table 1. Fifty-nine patients (14%) were discharged with favorable neurologic outcomes. They were more prone to liver cirrhosis, active cancer, and cardiac diagnosis at admission than patients with poor neurologic outcomes. Considering arrest characteristics, patients with favorable outcomes were more likely to have shockable rhythms, short resuscitation durations, presumed cardiac causes of arrest, and low GO-FAR scores than patients with poor neurologic outcomes. Median value of pre-arrest albumin level was 2.5 g/dL among patients with favorable outcomes. It was significantly different from that of patients with poor outcomes (2.4 g/dL, *p* < 0.01).

**Table 1:** Clinical characteristics of included patients in the derivation cohort.

Characteristics	Total (N = 419)	Favorable neurologic outcome (N = 59)	Poor neurologic outcome (N = 360)	*p*
**Demographics**				
Age (years)	64.0 (55.0–74.0)	60.0 (54.0–71.0)	65.0 (55.0–75.0)	0.104
Male	273 (65.2)	42 (71.2)	231 (64.2)	0.376
**Comorbidities**				
Hypertension	153 (36.5)	19 (32.2)	134 (37.2)	0.560
Diabetes mellitus	126 (30.1)	17 (28.8)	109 (30.3)	0.879
Coronary artery disease	58 (13.8)	8 (13.6)	50 (13.9)	1.000
Heart failure	106 (25.3)	20 (33.9)	86 (23.9)	0.108
Chronic pulmonary disease	42 (10.0)	5 (8.5)	37 (10.3)	0.817
Chronic kidney disease	66 (15.8)	8 (13.6)	58 (16.1)	0.704
Liver cirrhosis	52 (12.4)	2 (3.4)	50 (13.9)	0.019
Active cancer	147 (35.1)	12 (20.3)	135 (37.5)	0.012
**Diagnosis at admission**				
Cardiac	137 (32.7)	32 (54.2)	105 (29.2)	< 0.001
Other medical	206 (49.2)	11 (18.6)	195 (54.2)	< 0.001
Surgical	68 (16.2)	14 (23.7)	54 (15.0)	0.125
Trauma	3 (0.7)	0 (0.0)	3 (0.8)	1.000
**Characteristics of arrest**				
Witnessed	384 (91.6)	56 (94.9)	328 (91.1)	0.449
Shockable rhythm	77 (18.4)	28 (47.5)	49 (13.6)	< 0.001
Resuscitation duration (min)	8.0 (4.0–24.0)	4.0 (2.0–6.0)	10.0 (4.0–28.0)	< 0.001
Presumed cardiac cause	108 (25.8)	33 (55.9)	75 (20.8)	< 0.001
GO-FAR score	10.0 (2.0–18.0)	-8.0 (-1.0–4.0)	11.0 (4.0–18.0)	< 0.001
Pre-arrest albumin (g/dL)	2.5 (2.0–3.0)	2.5 (3.1–3.5)	2.4 (2.0–2.9)	< 0.001

Data are presented as n (%) or median with interquartile ranges.

*GO-FAR score* Good Outcome Following Attempted Resuscitation score.

Variables were examined using univariable analyses, and those with *p* values less than 0.1 were included in a multivariable model. Albumin quartiles were significantly associated with good neurologic outcomes (OR, 1.590; 95% CI 1.498–7.134; *p* = 0.003) along with shockable rhythm (*p* = 0.041), resuscitation duration (*p* < 0.001), presumed cardiac cause of arrest (*p* = 0.036), and GO-FAR score (*p* < 0.001) (Table 2).

**Table 2:** Multivariable logistic analysis for favorable neurologic outcome on discharge in the derivation cohort.

Variables	Multivariable		
	Adjusted OR	95% CI	*p*
Liver cirrhosis	0.209	0.040–1.100	0.065
Active cancer	0.906	0.312–2.634	0.856
Cardiac diagnosis	2.315	0.157–34.173	0.541
Other medical diagnosis	0.473	0.033–6.689	0.579
Surgical diagnosis	1.979	0.129–30.387	0.624
Shockable rhythm	2.495	1.036–6.006	0.041
Resuscitation duration (min)	0.890	0.843–0.939	< 0.001
Presumed cardiac cause	3.206	1.080–9.511	0.036
GO-FAR score	0.873	0.830–0.918	< 0.001
Albumin quartiles (g/dL)	1.590	1.082–2.337	0.018

*OR* odds ratio, *CI* confidence interval, *GO-FAR score* Good Outcome Following Attempted Resuscitation score.

Each albumin quartile was compared with the reference group Q1. There was a statistically significant association between good neurologic outcomes and albumin quartiles as a single variable (*p* for trend < 0.001), and the outcome of the Q4 group alone was significantly different from that of the Q1 group after adjustment (adjusted OR, 1.733; 95% CI 1.098–2.733; *p* for trend = 0.018) (Table 3). Based on this result, the quartiles were categorized into three ordinal scales (1, 0, and − 2) as described in the “Methods” section.

**Table 3:** Discharge with favorable neurologic outcome by albumin quartiles in the derivation cohort.

Quartiles	Total	Favorable outcome (%)	Crude OR (95% CI)	Adjusted OR (95% CI)
Q1 (< 2.1 g/dL)	109	6 (5.5%)	Reference	Reference^a^
Q2 (2.1–2.5 g/dL)	123	11 (8.9%)	1.686 (0.602–4.723)	0.966 (0.204–4.559)
Q3 (2.6–3.0 g/dL)	92	10 (10.9%)	1.447 (0.855–2.449)	1.030 (0.502–2.113)
Q4 (> 3.0 g/dL)	95	32 (33.7%)	2.058 (1.511–2.803)	1.733 (1.098–2.733)
*p* for trend			< 0.001	0.018

*OR* odds ratio, *CI* confidence interval, *GO-FAR score* Good Outcome Following Attempted Resuscitation score.

^a^Adjusted for liver cirrhosis, active cancer, diagnosis at admission, shockable rhythm, resuscitation duration, presumed cardiac cause, GO-FAR score.

### Model performance and validation

The albumin-added scores for patients in the derivation cohort were calculated by adding categorized albumin scores to the original GO-FAR scores. The improvement in prediction of neurologic outcome was evaluated by calculating the net reclassification index (NRI) and integrated discrimination improvement (IDI), in addition to AUROC comparison between the two models. The AUROCs of the original GO-FAR model and albumin-added model were 0.839 and 0.848, respectively. The difference between AUROCs was 0.008 (95% CI 0.001–0.016; *p* = 0.033). The NRI and IDI of the new model were 0.059 (95% CI − 0.037 to 0.119; *p* = 0.051) and 0.006 (95% CI 0.000–0.012, *p* = 0.049), respectively.

These results were also evaluated in the validation cohort. They were consistent with the results of the derivation cohort. In the validation cohort, the difference between AUROCs was 0.008 (95% CI 0.001–0.016; *p* = 0.034). The NRI and IDI of the new model were 0.072 (95% CI 0.013–0.139; *p* = 0.017) and 0.007 (95% CI 0.0010–0.013; *p* = 0.018), respectively (Table 4).

**Table 4:** Comparison of the discriminating performance for the poor neurologic outcome with and without the albumin quartiles by AUROC, NRI, and IDI in the derivation and validation cohorts.

Prediction model	AUROC (95% CI)	Difference of AUROC (95% CI)	NRI (95% CI)	IDI (95% CI)
**Derivation cohort**				
GO-FAR score	0.839 (0.792–0.887)			
GO-FAR score + albumin quartiles	0.848 (0.802–0.893)	0.008 (0.001–0.016)	0.059 (− 0.037 to 0.119)	0.006 (0.000–0.012)
*p*		0.033	0.051	0.049
**Validation cohort**				
GO-FAR score	0.791 (0.735–0.846)			
GO-FAR score + albumin quartiles	0.799 (0.745– 0.853)	0.008 (0.001–0.016)	0.072 (0.013–0.132)	0.007 (0.001–0.013)
*p*		0.034	0.017	0.018

*AUROC* area under the receiver operating characteristic curve, *NRI* net reclassification index, *IDI* integrated discrimination improvement, *CI* confidence interval, *GO-FAR score* Good Outcome Following Attempted Resuscitation score.

## Discussion

We evaluated the predictive value of pre-arrest albumin level and compared the predictive performances for favorable neurologic outcomes between the original GO-FAR model and the albumin-added model. We found that pre-arrest albumin level was independently associated with neurologic outcomes, and the predictive performance of the original GO-FAR model improved when the albumin level was combined with ordinal scale. These results were consistent with those of the validation cohort.

Recently, there have been several studies, which reported that low serum albumin level was associated with unfavorable neurologic outcomes in OHCA patients^16,18^. Kong et al. reported that lactate/albumin ratio showed better predictive performance for neurologic outcomes and survival to discharge after OHCA^19^. Apart from their clinical usefulness, serum albumin levels are not readily measured in OHCA patients. This is because it is difficult to obtain blood samples, and it takes approximately 2 h to identify the results. In contrast, laboratory tests to assess albumin levels are routinely performed among admitted patients; therefore, we can easily assess the results among IHCA patients. This is the first study investigating the association between pre-arrest serum albumin levels and neurologic outcomes of IHCA patients. Our results are also consistent with the data from other studies on the association between albumin levels and critical illnesses^14^.

Our study had a few limitations. First, almost all included patients (90.0% of the derivation cohort, 94.5% of the validation cohort) had hypoalbuminemia, i.e. their albumin levels were less than 3.5 g/dL. Considering its clinical importance, approximately half of the patients had clinically significant hypoalbuminemia (albumin levels < 2.5 g/dL)^20^. The included patients had undergone IHCA; thus, they had more comorbidities than healthy patients. Additionally, about 35% of the patients had active cancer, and cachexic patients were likely to have low albumin levels^21^. Nevertheless, there were significant associations between albumin levels and neurologic outcomes; therefore, it may be assumed that the results would be consistent even if a larger population is analyzed. Second, we could not identify if the patients were supplemented with albumin solutions. Most of the admitted hypoalbuminemic patients received albumin solutions especially when they were critically ill or in a state of shock. The albumin solutions could have been administered just before cardiac arrests. However, the measured albumin level was associated with outcomes regardless of replacement, suggesting that albumin replacement could cause favorable outcomes in IHCA patients. Third, there was only minimal improvement in the new albumin-added prediction model. Despite its statistical significance, improvement in AUROC was only 0.008. The improvement was also validated in the validation cohort, however, the study population was not divided randomly. Instead of randomization, we divided the study population by half according to a specific date. Although there were no statistically significant differences in baseline characteristics and the study outcome between the derivation and validation cohorts, the validation might have been influenced by selection bias (Supplementary Table S2).

We presented the NRI in the results section, and it was 5.9% and 7.2% in the derivation and validation cohorts, respectively. Even though we found the improvement in AUROC by adding the albumin level, the values are not very high. Its clinical implications will be very limited, and the albumin level cannot substitute for a variable in the GO-FAR score. On the other hand, it has been known that if established risk thresholds exist and treatment decisions are made based on risk categories, NRI which uses these categories can be useful^22^. Based on the calculated NRI we estimated that physicians can make more accurate decisions for approximately 17,500 patients annually who can be reclassified using the new prediction model. This may reduce the burden on societies and families^23^.

Discuss with patients and their families about the topic of do-not-resuscitation (DNR) in advance is essential to respect the wishes of the patient^24^. It is often difficult to precisely predict the outcomes of imminent resuscitation procedures, particularly in unpredicted, chaotic situations of cardio-pulmonary resuscitations. We focused on finding a pre-arrest stratification tool for helping physicians decide, importantly pre-arrest values that easily obtainable. As Ebell et al. mentioned in the original study that the GO-FAR score should be used in conjunction with clinical judgment, also the new prediction model can never be used in isolation, should rather be a part of a decision that considers the patient’s preferences^6^. Although there have been efforts to find an accurate predictor, other scores such as the Prognosis After Resuscitation score, the modified PAM index, and the Pre-Arrest Morbidity score were found to lose their accuracy in the validation’s studies^25,26^. On the contrary, GO-FAR score is a valid stratification tool for patients with IHCA, however, our study results showed only a minor improvement in the predictive performance of the GO-FAR score^27^. Therefore, future research requires the identification of more valuable biomarkers and the development of robust scoring systems for predicting the outcomes of patients with cardiac arrests with lower false positive rates.

## Conclusion

The adaptation of the GO-FAR score combining pre-arrest serum albumin levels showed better predictive accuracy than the original GO-FAR score for neurologic outcomes of IHCA patients. However, the improvement of predictive performance was modest.

## Supplementary Information

Supplementary Figure S1.

Supplementary Information.

## Data Availability

The data that analyzed during the current study are available on reasonable request from the corresponding author.

## Abbreviations

AUROC: Areas under receiver operating characteristic curve
CI: Confidence interval
CPC: Cerebral performance category
CPR: Cardiopulmonary resuscitation
GO-FAR: Good Outcome Following Attempted Resuscitation
IDI: Integrated discrimination improvement
IHCA: In-hospital cardiac arrest
MET: Medical emergency team
NRI: Net reclassification index
OHCA: Out-of-hospital cardiac arrest
OR: Odds ratio
