# Supplementary figures and images for: Predictive value of pre-arrest albumin level with GO-FAR score in patients with in-hospital cardiac arrest
Source: Sci Rep. 2021 May 20;11:10631. doi: 10.1038/s41598-021-90203-9 (PMC8138001; doi:10.1038/s41598-021-90203-9)

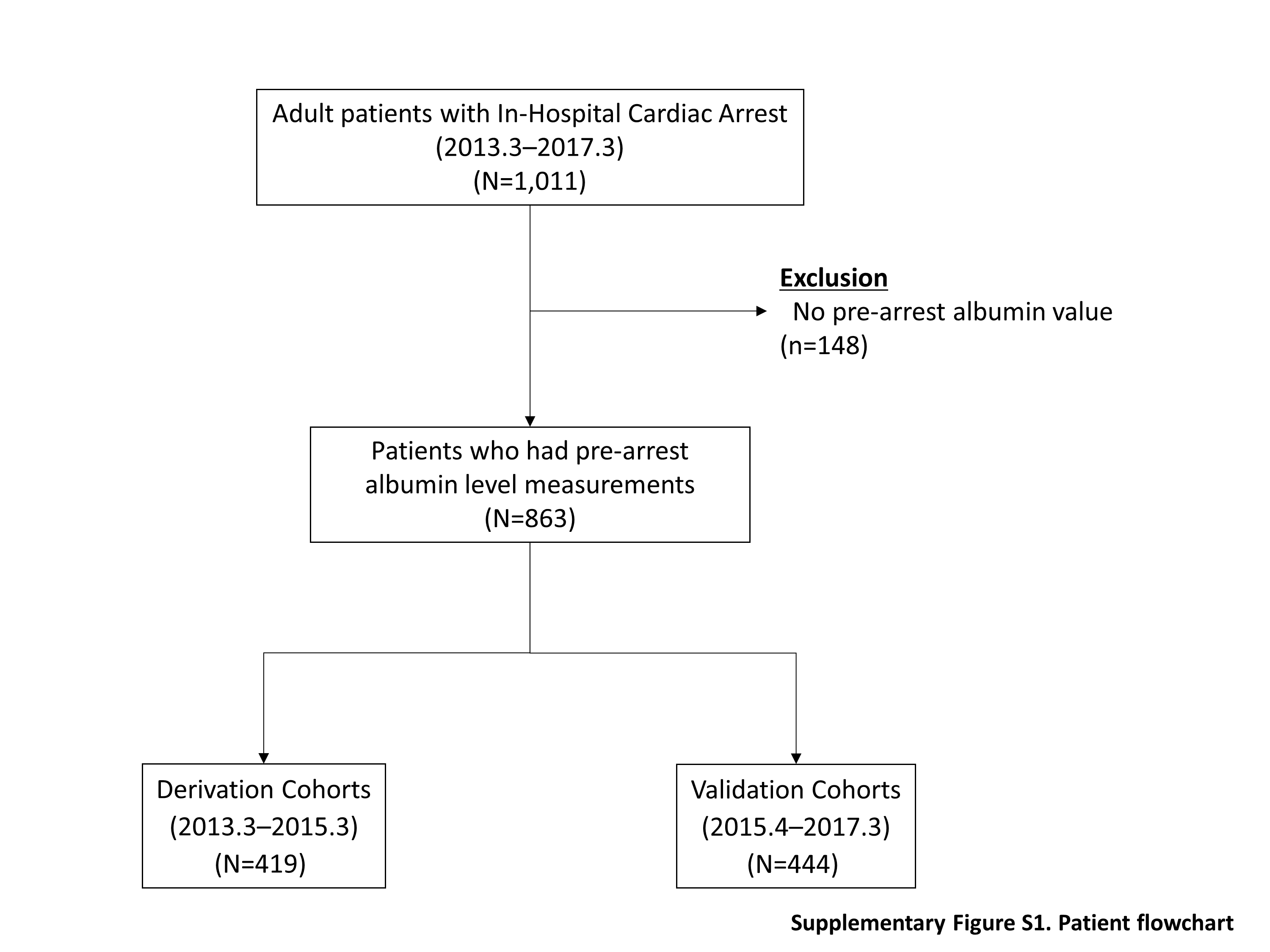

Supplement: Supplementary file 1 — Supplementary Figure S1. [file 41598_2021_90203_MOESM1_ESM.tif]
