# Supplementary material for: Predictive value of pre-arrest albumin level with GO-FAR score in patients with in-hospital cardiac arrest
Source: Sci Rep. 2021 May 20;11:10631. doi: 10.1038/s41598-021-90203-9 (PMC8138001; doi:10.1038/s41598-021-90203-9)
Supplement: Supplementary file 2 — Supplementary Information. [file 41598_2021_90203_MOESM2_ESM.docx]

Article

**Prognostic value of pre-arrest albumin level with GO-FAR score in patients with in-hospital cardiac arrest**

Seok-In Hong^1^, Youn-Jung Kim^1^, Yeon Joo Cho^2^, Jin Won Huh^3^, Sang Bum Hong^3^, Won Young Kim^1*^

^1^Department of Emergency Medicine, University of Ulsan College of Medicine, Asan Medical Centre, Seoul, Korea; ^2^Department of Emergency Medicine, Kyungpook National University School of Medicine, Daegu, Korea; ^3^Department of Pulmonary and Critical Care Medicine, University of Ulsan College of Medicine, Asan Medical Centre, Seoul, Korea;

* Correspondence to: Won Young Kim, Department of Emergency Medicine, University of Ulsan College of Medicine, Asan Medical Centre, Seoul 05505, Korea: Tel: +82-2-3010-3350, Fax: +82-2-3010-3360, Email: [wonpia73@naver.com](mailto:wonpia73@naver.com)

**Supplementary figure legends**

Supplementary Figure S1. Patient flowchart

**Supplementary Tables**

Supplementary Table S1. GO-FAR score variables

| Variables | Score |
| --- | --- |
| Neurologically intact at admission | -15 |
| Major trauma | 10 |
| Acute stroke | 8 |
| Metastatic or hematologic cancer | 7 |
| Septicemia | 7 |
| Medical non-cardiac diagnosis | 7 |
| Hepatic insufficiency | 6 |
| Admission from skilled nursing facility | 6 |
| Hypotension or hypoperfusion | 5 |
| Renal insufficiency including dialysis | 4 |
| Respiratory insufficiency | 4 |
| Pneumonia | 1 |
| Age (years) |  |
| 70–74 | 2 |
| 75–79 | 5 |
| 80–84 | 6 |
| ≥85 | 11 |

Supplementary Table S2. Comparison of baseline characteristics and the study outcome between the derivation and validation cohorts.

| Characteristics | Total  (N=863) | Derivation Cohort (N=419) | Validation Cohort (N=444) | *p* |
| --- | --- | --- | --- | --- |
| **Demographics** | | | | |
| Age (years) | 64.0 (54.0–74.0) | 64.0 (55.0–74.0) | 65.0 (53.0–74.0) | 0.633 |
| Male | 547 (63.4) | 273 (65.2) | 274 (61.7) | 0.322 |
| **Comorbidities** | | | | |
| Hypertension | 320 (37.1) | 153 (36.5) | 167 (37.6) | 0.778 |
| Diabetes mellitus | 278 (32.2) | 126 (30.1) | 152 (34.2) | 0.215 |
| Coronary artery disease | 123 (14.3) | 58 (13.8) | 65 (14.6) | 0.771 |
| Heart failure | 189 (21.9) | 106 (25.3) | 93 (20.9) | 0.121 |
| Chronic pulmonary disease | 87 (10.1) | 42 (10.0) | 45 (10.1) | 1.000 |
| Chronic kidney disease | 154 (17.8) | 66 (15.8) | 88 (19.8) | 0.131 |
| Liver cirrhosis | 106 (12.3) | 52 (12.4) | 54 (12.2) | 0.918 |
| Active cancer | 315 (36.5) | 147 (35.1) | 168 (37.8) | 0.437 |
| **Diagnosis at admission** | | | | |
| Cardiac | 277 (32.1) | 137 (32.7) | 140 (31.5) | 0.716 |
| Other medical | 427 (49.5) | 206 (49.2) | 221 (49.8) | 0.892 |
| Surgical | 142 (16.5) | 68 (16.2) | 74 (16.7) | 0.927 |
| Trauma | 6 (0.7) | 3 (0.7) | 3 (0.7) | 1.000 |
| **Characteristics of arrest** | | | | |
| Witnessed | 792 (91.8) | 384 (91.6) | 408 (91.9) | 0.902 |
| Shockable rhythm | 158 (18.3) | 77 (18.4) | 81 (18.2) | 1.000 |
| Resuscitation duration (min) | 8.0 (4.0–24.0) | 8.0 (4.0–24.0) | 8.0 (3.0–24.0) | 0.577 |
| Presumed cardiac cause | 226 (26.2) | 108 (25.8) | 118 (26.6) | 0.816 |
| GO-FAR score | 9.0 (1.0–17.0) | 10.0 (2.0–18.0) | 8.0 (1.0–16.0) | 0.184 |
| Pre-arrest albumin (g/dL) | 2.5 (2.0–3.0) | 2.5 (2.0–3.0) | 2.5 (2.0–2.9) | 0.505 |
| **Favorable neurologic outcome at discharge** | 127 (14.7%) | 59 (14.1) | 68 (15.3) | 0.632 |

Data are presented as n (%) or median with interquartile ranges.

Abbreviations: GO-FAR score, Good Outcome Following Attempted Resuscitation score

Supplementary Table S3. Clinical utility metrics of GO-FAR score, albumin level, and new model for favorable neurologic outcome in the derivation cohort

| Variables | AUROC  (95% CI) | *p* | Cut-off | Sensitivity (%) | Specificity (%) | PPV (%) | NPV (%) |
| --- | --- | --- | --- | --- | --- | --- | --- |
| Albumin  (continuous) | 0.718  (0.647–0.790) | <0.001 | 3.0 | 59.3 | 79.7 | 32.4 | 92.3 |
| Albumin  (quartiles) | 0.712  (0.641–0.784) | <0.001 | Quartile 4 | 54.2 | 82.5 | 33.7 | 91.7 |
| GO-FAR | 0.839  (0.792–0.887) | <0.001 | 5.0 | 79.7 | 71.7 | 31.5 | 95.6 |

Abbreviations: GO-FAR score, Good Outcome Following Attempted Resuscitation score; AUROC, area under the receiver operating characteristic curve; CI, confidence interval; PPV, positive predictive value; NPV, negative predictive value
